# Surface characterization and *in vivo* evaluation of laser sintered and machined implants followed by resorbable-blasting media process: A study in sheep

**DOI:** 10.4317/medoral.20946

**Published:** 2016-01-31

**Authors:** Michelle Bowers, Daniel Yoo, Charles Marin, Luiz Gil, Nour Shabaka, Matt Goldstein, Malvin Janal, Nick Tovar, Ronaldo Hirata, Estevam Bonfante, Paulo Coelho

**Affiliations:** 1 Department of Biomaterials and Biomimetics, New York University College of Dentistry, New York, USA; 2 Department of Oral Surgery, Federal University of Santa Catarina, Florianópolis SC, Brazil; 3 New York University Abu Dhabi, Abu Dhabi, United Arab Emirates; 4 Department of Epidemiology, New York University College of Dentistry, New York, USA; 5 Posgraduated program in Dentistry - Unigranrio University, School of Health Sciences, Duque de Caxias RJ, Brazil

## Abstract

**Background:**

This study aimed to compare the histomorphometric and histological bone response to laser-sintered implants followed by resorbable-blasting media (RBM) process relative to standard machined/RBM surface treated implants.

**Material and Methods:**

Six male sheep (n=6) received 2 Ti-6Al-4V implants (1 per surface) in each side of the mandible for 6 weeks *in vivo*. The histomorphometric parameters bone-implant contact (BIC) and bone area fraction occupancy (BAFO) were evaluated.

**Results:**

Optical interferometry revealed higher Sa and Sq values for the laser-sintered/RBM surface in relation to standard/RBM implants. No significant differences in BIC were observed between the two groups (*p*>0.2), but significantly higher BAFO was observed for standard/RBM implants (*p*<0.01).

**Conclusions:**

The present study demonstrated that both surfaces were biocompatible and osseoconductive, and the combination of laser sintering and RBM has no advantage over the standard machined implants with subsequent RBM.

**Key words:**Dental implants, osseointegration, resorbable- blasting media, sheep, in vivo.

## Introduction

Current research has progressed towards implant design modifications and improvements of early host-implant tissue response and reducing treatment time (1). The potential benefits include faster oral rehabilitation from surgery to prosthetic restoration, and better performance in low quality bone regions compared to standard classic protocols (1-3). Multiple design alterations, initially implant surface surface, have been investigated and attempted to analyze their effects upon function and survival within the host (1-9).

The methodology for implant surface modification relies upon the fact that the initial interaction point of the implant body involves the host tissue which may have widespread implications in bone healing and intimate apposition with the device (8). Despite the large number of possible modifications, previous publications have generally reported rough surfaces (compared to smooth surfaces) and surface chemistry (Ca-P-based bioceramic additions over machined surfaces) may favorably impact the early host-implant response (1,5,6,10-12). Implant surface texturing is typically completed during the post-fabrication process by milling to its desired macro-design (13-15). Surface roughness can be customized through its fabrication method, i.e. laser metal sintering (16-18).

Laser metal sintering is based on rapid prototyping, in which the compiled computer-aided design is constructed via a metal forming procedure with a high-power laser beam focused onto a metal powder bed and programmed for fusing particles to create a thin metal layer. The process continues until the progression of layer apposition completes the final 3D shape of the completed implant device (13-18). The final surface is marked as porous with functionally graded structures and an observed porosity gradient perpendicular to the implant long axis (16,17,19). Controlled surface porosity and the core materials may be selected to fit the implant’s intended purpose. In addition, the laser sintering process has been shown to produce a repeated porous pattern with an associated interconnected pore network, which may potentially improve overall osseointegration (13-18). The graded structure has also been claimed to decrease the discrepancy between the elastic modulus of titanium and that of the surrounding bone and form a favorable reduction in the interface stress (13-17). Laser sintering is suitable for a wide range of applications in the production of temporary or permanent implantable devices, particularly for designs required to construct an implant’s structural and biological function (13).

In vivo evaluation of laser sintered implants compared with AB/AE moderade rough surface showed higher values of BIC (bone-to-implant contact) and BAFO (bone area fraction occupied) parameters with statistical significance for the first week was found, similar values were found for 3 and 6 weeks. When torque to interface failure was measured higher values for sintered group for one and six weeks were found. Its advocated the implant micro-design achieved by laser-sintered may have provide an homogeneous and larger surface area for blood clot retention improving osseointegration process in its early stages (19). After the well described moderate rough surfaces (5) the Ca-P incorporation on the implant device (i.e. surface chemistry alteration) has also shown to improve osseoconductivity. Resorbable blasting media (RBM) is one of the available methods to integrate Ca-P particles at a reduced amount on and into the implant surface. A previous study comparing five different implant systems showed that RBM surface presented similar bone-to-implant contact (BIC) when compared to others at 3 and 6 weeks, as well as maintain its bone elastic modulus and hardness values over the time (20). Despite the positive results in existing *in vitro* and *in vivo* data on laser metal sintering implants, subsequent surface treatment in these devices has not been yet assessed. The aim of present study is evaluate the effect of laser sintering followed by RBM process compared to standard machined implants, followed by a similar RBM process. The current hypothesis is the complex surface topography from the laser sintering combined to RBM would improve the overall histomorphometric parameters compared with the machined/RBM implants.

## Material and Methods

The implants utilized in this study were Ti-6Al-4V screw type, tapered implants with 3.5 mm of diameter and 10 mm in length provided by the manufacturer (Adin, Afula, Israel). A total of 24 implants were used and divided into two groups according to fabrication process: standard machined implants with RBM blasted surface (Osseofix) (control) and laser-sintered implants that were also subsequently RBM blasted (experimental) (n=12 per group). Two additional implants were used for surface characterization.

- Surface Characterization

Surface characterization was carried out with three different methods. The first method involved scanning electron microscopy (SEM) (Zeiss, Oberkochen, Germany) that was performed at various magnifications under an acceleration voltage of 20 kV to characterize the differences in surface topography within each group (n=1 per group).

The second method was employed to determine the roughness parameters by optical interferometry (IFM) (Zeiss, Oberkochen, Germany; PhaseView 2.5, Palaiseau, France). One implant of each surface was evaluated at the flat region of the implant cutting edges (five measurements per implant) in terms of Sa (arithmetic average high deviation), Sq (root mean square). A filter size of 100 x 100 µm2 was utilized.

- Implantation Procedures

All animal experiments were conducted in accordance with the ethical approval The study was approved by the Ethics Committee for Animal Research at the E´ cole Nationale Véetérinaire d´Alfort (Maisons-Alfort, Val-de-Marne, France). Six Finnish Dorset cross-bred sheep (each weighing approximately 70 kg) were utilized for this study. The implants were inserted into the sheep mandible base of each animal. Prior to surgery, the mandibular regions were shaved using aseptic procedures. The animals were then monitored continuously for heart rate, oxygen saturation, respiratory rate, temperature, and tissue coloration prior to the complete shaving of the intended surgical site. The relevant adjacent areas were also accessed prior to the application of a povidone–iodine solution.

Monitoring was then transferred to an automated system and the animal draped aseptically. Anesthesia was induced with sodium pentothal (15-20 mg/kg) in Normasol solution in the jugular vein. Anesthesia was maintained with isoflurane (1.5-3%) in O2/N2O (50/50). Preoperatively and postoperatively, 500 mg cefazolin was administered intravenously. Throughout anesthesia, body temperature was maintained with a circulating hot water blanket placed underneath the sheep. Vital signs were intermittently monitored with electrocardiography, end-tidal CO2, and SpO2. The surgical procedure was performed at 6 weeks prior to euthanasia by means of a 10-cm extra oral incision located 2 cm distally from the most distal position of the masseter. After mandibular bone exposure, the osteotomy was prepared using the drills provided by the manufacturer. Each animal received two implants of both control and experimental groups (n=4 implants per animal). Each side of the mandible receive one implant from each group, that were randomly placed in the sites in a proximal-to-distal order at 2-cm intervals from the adjacent implant centers.

The implant groups were interpolated as a function of implantation site to minimize site bias throughout the study. Postoperative antibiotic and anti-inflammatory medications included a single dose of benzylpenicillin benzathine (20,000 IU/kg) intramuscularly and ketoprofen 1% (1 mL/5 kg). The sheep were euthanized by anesthesia overdose, and the mandibles were retrieved by sharp dissection. The soft tissue was removed using surgical blades, and an initial clinical evaluation was performed to determine implant stability. If an implant was clinically unstable, it was excluded from the study.

- Histological Preparation and Histomorphometry

Each experimental implant group was processed for histological and histomorphometric evaluation via progressive dehydration in alcohol and methyl salicylate before final embedding in methylmethacrylate (MMA). Standard non-decalcified histological sections were prepared for each implant sample according to standardized methodology (21). The samples were first sectioned along the implant´s long axis with a slow speed precision diamond saw (Isomet 2000, Buehler Ltd., Lake Bluff, IL, USA) as slices of ~300 µm thickness. Each tissue section was glued to an acrylic plate with a photo labile acrylate-based adhesive (Technovit 7210 VLC adhesive, Heraeus Kulzer GMBH, Wehrheim, Germany) prior to grinding and polishing under abundant water irrigation with a series of silicon carbide (SiC) abrasive papers (400, 600, 800, and 1200) (Metaserv 3000, Buehler Ltd., Lake Bluff, IL, USA) to a final thickness of 70 µm. The finalized sections were then stained with Stevenel´s Blue and Van Gieson´s Picro Fuschin (SVG) stains.

Histologic observations and images were obtained using an automated slide scanning system and specialized computer software (Aperio Technologies, Vista, CA, USA). The bone-to-implant contact (BIC) was determined by means of a computer software (ImageJ, NIH, Bethesda, MD). The regions of bone-to-implant contact along the implant perimeter were subtracted from the total implant perimeter, and calculations were performed to determine the BIC. The bone area fraction occupied (BAFO) between threads in trabecular bone regions was determined by means of computer software (ImageJ, NIH, Bethesda, MD). The areas occupied by bone were subtracted from the total area between threads, and calculations were performed to determine the BAFO (reported in percentage values of bone area fraction occupied)

- Statistical Analysis

All histomorphometric data is presented as mean values with their corresponding 95% confidence intervals (95% CI), while the optical interferometry results were displayed with standard deviation (SD). The collected %BIC and %BAFO data were utilized to generate a general linear ANOVA model (NCSS LLC) with the level of significance set at *p*<0.05. The independent variable analyzed was the implant type.

## Results

- Animal procedure

The surgical procedure surgical and post-operatory period was uneventful. No implants were excluded from this study after samples retrieval due to clinical instability.

- Surface Characterization

**Figure 1:**
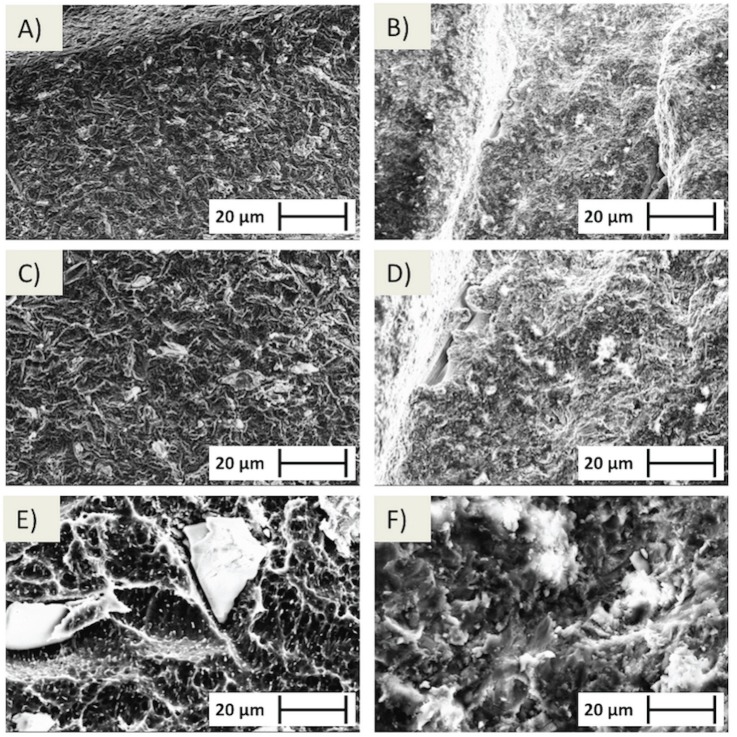
SEM images of standard (A, C, and E) and laser-sintered (B, D, and F) obtained under progressively higher magnification. Note RBM media inclusion within surface for both groups.

**Figure 2:**
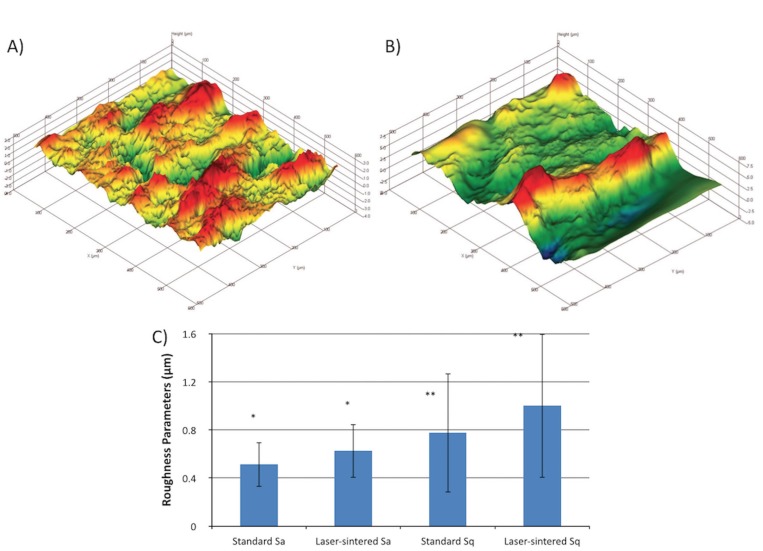
(A, B) Representative IFM reconstruction images (filter size of 100 x 100 µm2) of the standard and laser-sintered implants, respectively. (C) Statistical summary (mean ± SD) for surface roughness parameters, Sa and Sq, for each implant group. Note that the asterisks represent statistically homogenous groups.

Scanning electron micrographs (SEM) of both implant surfaces at different magnifications showing the inclusion of RBM for standart/RBM and laser-sintered/RBM are presented in figure 1. Three-dimensional IFM reconstructions (100 µm x 100 µm) showing remarkable differences between surface topography of control and experimental groups (Fig. 2A,B). Values of Sa and Sq are presented in figure 2C. Control and experimental groups presented statistical difference for both parameters measured (Sa and Sq) with higher values for experimental group (laser sintered/RBM) (*p*<0.05).

- Histomorphometric Parameters

**Figure 3:**
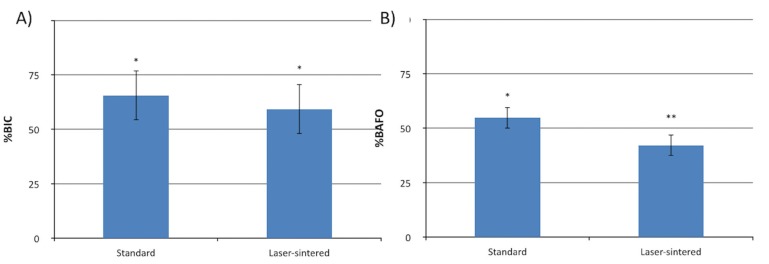
Statistical summary (mean ± 95% CI) for %BIC and %BAFO for standard and laser-sintered implants considering surface treatment at 6 weeks *in vivo*. Note that the asterisks represent statistically homogenous groups.

 No significant differences for BIC were observed between the standard/RBM and laser-sintered/RBM implant groups (*p*>0.2; Fig. 3A). However, the same pattern was not observed for BAFO, where standard/RBM implants presented significantly higher bone formation over that of laser-sintered/RBM implants (*p*<0.01; Fig. 3B).

- General Histological Observations

**Figure 4:**
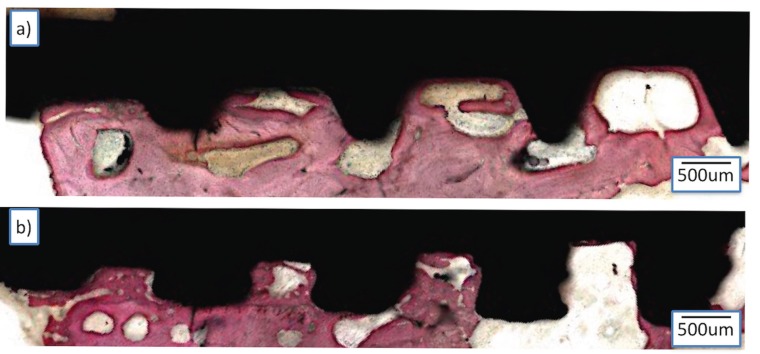
Histological sections showing bone healing around the standard (A) and laser-sintered (B) implant groups at 6 weeks post-implantation. Bars represent 200 µm.

Nondecalcified sample processing revealed intimate bone contact with all implant surfaces at cortical and trabecular bone regions. For both surfaces osteonic structures were observed in close contact of implant surface (arrows) demonstrating mature and well organized bone structure even in healing chambers formed by osteotomy diameter and implant macro-design. Bone formation was observed in contact with the implant away from the osteotomy line showing the intramembranous-like bone formation initiating from the implant surface after 6 weeks *in vivo* (asterisk) (Fig. 4).

## Discussion

Chemical and topographical surface modifications have earned extensive interest due to promising results shown in *in vitro* and *in vivo* relative to their commercial predecessors (1,5,6,22-31). The implant surface modification has evolved from as-machined, smooth surfaces to microscopically moderately roughened surfaces that have shown to enhance bone healing after the placement of implants (1,10,32). Laser sintered implants are classified as moderately rough range, proving to be an effective method to produce an osseoconductive surface (19,33-37). {Witek, 2012 #21}{Witek, 2012 #21}Subsequent surface chemistry modifications such as the incorporation of bioactive ceramics have long been the focus of investigations as a positive factor for improved early bone healing. However, considering that surface chemistry modifications typically involve changes in surface topography (25), it is still unclear whether resulting topography changes alone and/or the combination with chemical modifications leads to improved osseointegration (38).

The chemical modification of the surface after laser sintering, as the Ca-P incorporation after RBM process performed in the present study, could hypothetically improve histomorphometric results when compared to standard/RBM implants. Although Sa values reached statistically difference between standard/RBM (0.51 ± 0.19 µm) and laser-sintered/RBM (0.78 ± 0.49 µm) groups, both are classified as moderate rough surfaces. The data of present study showed an decrease of roughness parameters (Sa and Sq) when laser-sintered implants are processed by means of RBM, producing minimally rough surface. However, multiple processing variables appear to provide an effect on the finished surface topography of laser-sintered implants and probably on their osteogenic properties, i.e. the power rating of the laser, beam focus diameter, scanning speed, average particle size of the initiating material powder, atmospheric conditions, and others (39). Also, besides the sintering protocol, the combination of laser sintering and RBM could have affected overall Sa values. Marin *et al*. (40) evaluated Sa for an AB/AE, RBM/acid etched (RBMa) and a hybrid implant (AB/AE + RBMa). AB/AE implants showed higher Sa when compared to others, with the hybrid device reaching an intermediate value.

For Histomorphometric parameters evaluated in this study, only BAFO values were significantly different at 6 weeks *in vivo* with the standard/RBM group presenting higher overall values compared to those of laser-sintered/RBM implants. Previous studies have hypothesized that the three-dimensional surface configuration of the laser-sintered implant potentially produced a larger exposed surface area during the early wound healing cascade along with increased blood clot retention compared to the control implant group (19). BIC results showed no significant differences between the experimental and control groups. As such, the present scope of this study did not involve multiple and earlier time-points, which may have excluded the detection of early bone responses within the host.

Current literature have also reported no significant differences between groups by 6 weeks post-implantation as early bone healing may no longer be the dominant process in play at this point (19,20). Since secondary stability of the implanted device is one of the primary goals of design research, BIC and other related interfacial statistics may be the more vital histomorphometric category. Thus, despite the observed increase in BAFO within the standard/RBM group compared with laser-sintered/RBM group, the lack of statistically significant differences for BIC values could indicate the need for additional comparison studies between these two groups at early time-points in order to include the host early bone response. The general results from this study show that both implants investigated are biocompatible and osseoconductive, resulting in a similar bone-healing pattern at cortical and trabecular bone in close contact with the implant surface. The rationale of testing the association between laser sintering and RBM surface was to provide a rougher surface due to laser sintering, when compared to standard machined process, combined with the presence of Ca-P achieved with subsequent RBM treatment. However, our proposed hypothesis, when laser-sintered/RBM implants would enhance the overall histomorphometric parameters in comparison to the standard/RBM implant surface was not accepted due to the absence of significant differences in BIC values and negative effect on BAFO results.

The present study is limited in scope from a temporal perspective and requires additional investigation for the proper elucidation of laser-sintered/RBM implant materials and their effects within a sheep model. In addition to earlier time-points for observing the initial host wound and bone healing response, future studies with variations in specific processing parameters and surface physic-chemical characterization are warranted. These investigations would also provide substantial information by biomechanical (torque testing) (19), nanomechanical (nanoindentation) (41), and even the underlying genetic/molecular processes (bone markers) (42) corresponding to the observed tissue effects. Despite the utilization of several animal species, including sheep, in implant materials testing, variations in the innate remodeling rate must be considered when conducting *in vivo* studies of this nature (43).

## Conclusion

Based on the results observed in our investigation, both surfaces were biocompatible and osseoconductive, and the combination of laser sintering and RBM has no advantage over the standard machined implants with subsequent RBM.
